# The migration of South African emergency care practitioners to the Middle East

**DOI:** 10.4102/hsag.v30i0.2788

**Published:** 2025-02-28

**Authors:** Siyanda I. Mthombeni, Craig Vincent-Lambert

**Affiliations:** 1 Department of Emergency Medical Care, Faculty of Health Sciences, University of Johannesburg, Johannesburg, South Africa

**Keywords:** migration, Emergency Care Practitioner, South Africa, Middle East, employment, push factor, pull factor

## Abstract

**Background:**

The loss of healthcare professionals because of migration remains a challenge for low- and middle-income countries. South African Emergency Care Practitioners (ECPs) are no exception with many choosing to leave South Africa (SA) to work in the Middle East (ME).

**Aim:**

To investigate and describe the push and pull factors that are influencing ECPs to leave SA and work in the ME.

**Setting:**

The study took place in the ME in an online setting.

**Methods:**

A prospective mixed-method sequential design was adopted that made use of a purposively designed, pre-piloted, online questionnaire followed by a number of one-on-one interviews with a purposefully selected sample of participants to explore push and pull factors that influenced their decision to leave SA to work in the ME.

**Results:**

The increased cost of living, low salaries, poor and unsafe working environments, and being undervalued by management in SA were the main push factors that emerged. Better pay, improved safety and security and a better quality of life in the ME were identified as core pull factors that are drawing South African ECPs to the ME.

**Conclusion:**

To stem the ongoing exodus of ECPs, significant work has to be done to improve local salaries, working conditions and the overall job satisfaction experienced by operational ECPs in SA.

**Contribution:**

Understanding why ECPs are leaving the country, and in particular why they choose the ME as their employment destination can assist Emergency Medical Services (EMS) managers to implement measures that aid in the retention of these highly skilled healthcare professionals.

## Introduction

The loss of healthcare professionals because of economic migration has been a longstanding concern for many low- and middle-income countries (LMICs) (Joshi et al. 2023; Toyin-Thomas et al. 2023). This has resulted in a severe shortage of skilled healthcare workers (HCW) to cater for the population needs of LMICs. South African HCWs in particular remain globally competitive because of the high standard of health professions education and training they receive (Govender 2024). This, and their exposure to critical cases, renders South African HCWs prime candidates for recruitment by international role players from high-income countries (HICs) around the world.

While, from the researchers perspective, leaving one’s country to work and live abroad may come with significant personal benefits, the ongoing exodus of experienced healthcare professionals places an additional strain on the already overburdened healthcare systems of the countries they leave behind. Consequently, the loss of skilled and experienced staff because of migration should be seen as a shared concern for all as this hampers LMICs such as South Africa (SA) from achieving their Sustainable Development Goals (Stats SA 2023). In this context, it is unsurprising that several local and international studies have investigated the factors that contribute to HCWs’ decisions to leave their home country and seek employment elsewhere. Researchers have classified these as ‘push’ and ‘pull’ factors (Castro-Palaganas et al. 2017; Govender et al. 2012; Toyin-Thomas et al. 2023). Push factors are things that cause HCWs to leave their country, whereas the pull factors are things in the destination country that attract HCWs to accept employment offers abroad. Some studies have noted that push factors are often stronger than the pull factors (Bidwell et al. 2014; Reardon & George 2014). The major push factors that have been identified among different cadres of South African HCWs included low wages, poor and frustrating working conditions, job uncertainty and the high crime rate in SA (Govender et al. 2012; Mlambo & Adetiba 2017; Reardon & George 2014; Toyin-Thomas et al. 2023).

The professionalisation and restructuring of the pre-hospital emergency care education and training in SA has seen the emergence of highly skilled pre-hospital emergency care providers, known locally as Emergency Care Practitioners (ECPs) (Sobuwa & Christopher 2019; South African National Department of Health 2017; Vincent-Lambert, Bezuidenhout & Vuuren 2014). Emergency Care Practitioners are healthcare professionals that have completed clinically oriented 4-year professional bachelor degrees in emergency medical care. Emergency care practitioner graduates register with the Health Professions Council of South Africa (HPCSA) as independent practitioners. Their clinical skills and extensive scope of practice in emergency and critical care have caused South African ECPs to become a highly sought-after cadre of pre-hospital emergency care provider, both locally and abroad. As a consequence, South African Emergency Medical Services (EMS) continue to lose ECPs to aggressive recruitment drives by foreign governments and private EMS organisations, particularly from the Middle East (ME) region.

The ME region is located more or less between Africa and Asia. It includes countries such as the United Arab Emirates (UAE), Qatar, the Kingdom of Saudi Arabia (KSA), Oman, Jordan, Bahrain, Kuwait, Egypt, Iraq, Palestine, Yemen, Syria, Israel, Lebanon, Cyprus, Turkey and Iran. These role players offer lucrative benefits and opportunities that have seen many South African ECPs accepting offers to work abroad shortly after qualifying. This steady exodus of ECPs creates staff shortages that impact the ability of local EMS to provide advanced life support (ALS) and high-quality pre-hospital emergency care services in many regions of the country.

The majority of local and international literature describes the migration of in-hospital staff such as doctors, nurses, dentists and pharmacists (Bezuidenhout et al. 2009; George, Atujuna & Gow 2013; Mlambo & Adetiba 2017; Reardon & George 2014; Saluja et al. 2020). Less literature exists that deals with the migration of South African pre-hospital emergency care workers such as ECPs (Binks 2011; Gangaram 2015; Govender et al. 2012). This prompted us to conduct this study to attempt to gain a deeper understanding of the factors that are associated with the migration of qualified ECPs from SA to the ME. A better understanding of these factors can assist EMS managers in SA to design and implement measures that may assist in retaining their ECP graduates within the country.

## Research methods and design

### Design

In order to frame and contextualise the study, the researcher started by conducting a comprehensive literature review to identify the push and pull factors that were specific to pre-hospital providers. These factors were pre-determined and not emergent for the questionnaire; however, there was still an option to add other factors that the respondents felt influenced them to migrate to the ME. A prospective, mixed-method sequential design consisting of two phases was adopted. In the first quantitative phase, we made use of a purposively designed self-developed online questionnaire. The questionnaire was pre-piloted and consisted of five sections that focussed on gathering information about demographics, employment in SA and the ME, pushing factors from SA and pulling factors from the ME. Invites and links to the questionnaire were distributed via social media platforms with a message of forwarding the link to ECPs working in the ME. The link to the questionnaire was opened for 3 months, and reminders were sent every 2 weeks during this time.

Phase Two of the study consisted of a number of one-on-one interviews with a sample of purposively selected information-rich participants who indicated that one or more of the top four push and pull factors found after the analysis of the questionnaire influenced them to leave SA and choose the ME as their employment destination. Semi-structured, pre-piloted open-ended questions were generated by the researcher to guide and form part of the discussion during the interviews.

### Sample

To complete the online questionnaire, respondents must have been a SA-qualified ECP with at least 1 year’s clinical experience both in SA and in the ME. While our target population may be considered to include all SA-qualified ECPs who were working in the ME at the time of the study, determining the exact number of these individuals proved to be difficult. For, at the time of the study, there were 731 ECPs registered with the HPCSA; however, the HPCSA I-register does not show if they are operational nor where they are working. By the time the questionnaire closed, a final sample of 87 respondents completed the survey questionnaire. However, analysis of the responses showed that, of these 32 did not meet the inclusion criteria and only 55 completed questionnaires were available for analysis. Subsequently, 28 invitational emails were sent out to the pool of respondents who showed interest for phase two of the study and only 13 responded to the invites. Data saturation was seen to have occurred after the ninth interview.

### Data analysis

Responses to each of the questions in the questionnaire were exported to an electronic spreadsheet. This allowed us to identify selected demographic data including the main push and pull factors that contributed to the respondents’ deciding to leave SA and work in the ME. A Statistical Package for the Social Sciences (version 26.0, SPSS Science, Chicago, US) was used to assist with the descriptive analysis of the raw data. For the interviews, thematic analysis was used to analyse and code data in the form of the transcripts. A qualitative data analysis software application (ATLAS.ti Scientific Software Development GmbH, version 8) was used for this process, and it led to the identification of emerging themes and sub-themes.

### Ethical considerations

Ethical approval for this study was obtained from the University of Johannesburg, Faculty of Health Sciences Human Research Committee (No. REC-714-2020). Informed consent was obtained from all respondents and participants before they could gain access to the online questionnaire and/or participate in one of the follow-up interviews. Respondents were free to withdraw from the online questionnaire at any time before the final responses were submitted. No respondents or participants are identified in any of the reported data. All data were treated as strictly confidential and kept in a password-protected file.

## Results

Table 1 presents the descriptive characteristics of our respondents’ who were working in the ME. Unmarried males with less than 4 years’ work experience in SA were found to be the group that is prone to leave SA and migrate to the ME.

**TABLE 1:** Respondents characteristics.

Demographics	Frequency	%
**Gender**		
Male	38	69.1
Female	17	30.9
**Age groups (years)**		
26–30	14	25.5
31–35	22	40.0
36–40	9	16.4
**Marital status**		
Non-married (single, divorced, spousal relationship)	32	58.2
Married	23	41.8
**Work experience in SA**		
< 4 years	41	74.6
> 4 years	14	25.4
**Work experience in ME**		
< 2 years	24	43.6
> 2 years	31	56.4

Mthombeni, S.I., 2024, ‘Factors influencing the migration of South African emergency care practitioners to the Middle East’, Masters thesis, University of Johannesburg, Johannesburg, viewed n.d., from. https://hdl.handle.net/10210/511825

SA, South Africa; ME, Middle East.

### Push and pull factors

Push and pull factors that were selected by our respondents were ranked by frequency of responses and ordered from most influential to least influential. Table 2 presents the push factors that influenced our respondents to look for employment outside of SA. Low salaries in SA, the desire to gain international EMS experience, poor working conditions, and management in the local EMS in SA were found to be the leading push factors.

**TABLE 2:** Push factors (*N* = 55).

Push factors	Proportion	%
Low take-home salaries in SA	48/55	87.3
Lack of opportunity to gain international EMS experience	31/55	56.4
Poor working environment and conditions	25/55	45.5
Poor management in the workplace	22/55	40.0
Unsafe workplace	21/55	38.2
Unstable economic and political conditions	19/55	34.5
Poor quality of life	16/55	29.1
Lack of infrastructure and resources	9/55	16.4
Lack of educational and career development opportunities	9/55	16.4
Frustrations with the health regulatory body (HPCSA)	5/55	9.1

Mthombeni, S.I., 2024, ‘Factors influencing the migration of South African emergency care practitioners to the Middle East’, Masters thesis, University of Johannesburg, Johannesburg, viewed n.d., from https://hdl.handle.net/10210/511825

SA, South Africa; EMS, Emergency Medical Services; HPCSA, Health Professions Council of South Africa.

Table 3 presents the pull factors that influenced our respondents to choose the ME as their employment destination. Higher wages, the prospect of gaining international EMS experience, a generally safer working environment with a better quality of life were the leading pull factors from the ME.

**TABLE 3:** Pull factors (*N* = 55).

Pull factor	Proportion	%
Higher wages	55/55	100.0
Safer working environment	34/55	61.8
Gaining international EMS experience	35/55	63.6
Better quality of life	32/55	58.1
Better working conditions and environment	25/55	45.5
Adequate infrastructure and resources	24/55	43.6
Stable economic and political conditions	22/55	40.0
Better educational and career advancement opportunities	14/55	25.5
More efficient health regulation authority	13/55	23.6

Mthombeni, S.I., 2024, ‘Factors influencing the migration of South African emergency care practitioners to the Middle East’, Masters thesis, University of Johannesburg, Johannesburg, viewed n.d., from https://hdl.handle.net/10210/511825

EMS, Emergency Medical Services.

The top four leading push and pull factors that were found from the questionnaire were explored through one-on-one interviews. The transcripts of these interviews were analysed through the development of codes, and these codes were further collapsed to create themes and sub-themes. Table 4 presents the themes and sub-themes that emerged from the analysis of the interviews together with the selected quotations from our participants.

**TABLE 4:** Core themes and sub-themes emerging from interviews.

Theme	Push sub-themes	Pull sub-themes
1. Financial considerations	1.1. Salaries that are not keeping up with the increasing cost of living in SA. ‘[*T*]he salaries or the modes of compensation do not actually make it [possible] for us to stay in SA.’ (Participant 03, male, public sector) 1.2. High tax and deductions resulting in low take-home pay in SA. ‘[*I*]n SA … they’re going to ask you 40% tax’ (Participant 05, male, private sector) 1.3. The ECP qualification not adequately recognised by the Occupation Specific Dispensation (OSD) in SA. ‘… OSD should be reviewed, especially in favour of the current development in EMS and emergency medical care qualification structure’ (Participant 01, male, public sector)	1.1. Higher salaries with added incentives in the ME. ‘[*T*]he UAE where it offered as I said a conclusive package. Where you come and stay and you have all these amenities [*incentives*] to benefit from.’ (Participant 04, male, public sector) 1.2. Less or no tax and deductions when working in the ME. ‘… I was getting paid double of what I was going to get paid in South Africa, but without tax.’ (Participant 02, male, private sector) ‘[*T*]he fact that you don’t pay tax … it just means you are having more money …’ (Participant 07, male, private sector)
2. Desire to gain international experience	2.1. Limited opportunity for international exposure to other EMS systems. ‘[*T*]he environment [*in SA*] was no longer serving its purpose for me personally.’ (Participant 01, male, public sector) 2.2. A desire to see other systems and implement changes in SA EMS. ‘We have been exposed to the solutions, but it is now for us as agents of change to bring those solutions and ‘… [*T*]o change and implement it according to the context of SA.’ (Participant 09, male, public sector)	2.1. Opportunity to gain international experience. ‘[*T*]o get international experience about working in another system, a more developed system. A more robust, a system that has key components in place that are missing in South African EMS.’ (Participant 09, male, public sector) 2.2. Increased employment opportunities internationally for SA-qualified ECPs. ‘ECPs currently are a commodity …’ (Participant 05, male, private sector) ‘… South Africans are extremely highly qualified medical practitioners and they are well rounded.’ (Participant 05, male, private sector) 2.3. Prospect of learning and gaining experience from a developed system/country. ‘[*G*]oing out there and see some of the best EMSs in the world, or some of the worst EMSs in the world.’ (Participant 04, male, public sector) ‘… [*W*]e can learn a lot from not only clinical, but also from the operations of the EMS on how they actually run the EMS, are they making things much easier and better?’ (Participant 03, male, public sector)
3. Working conditions and environment	3.1. Unbearable workload as a result of the shortage of ECPs. ‘When I qualified there weren’t any ECPs around, I was the only operational ECP. So I worked hard and I work a lot and it got really very tough …’ (Participant 06, female, private sector) 3.2. Increased frustrations with the SA EMS. ‘I was frustrated, and I could see the direction that this thing [*organisation*] is going to give me headaches.’ (Participant 01, male, public sector) 3.3. Unsafe working and living conditions in SA. ‘[*F*]or the last four or five years now paramedic attack it’s an ongoing issue and it’s not safe anymore.’ (Participant 01, male, public sector)	3.1. Safer working and living environment in the ME. ‘ [*C*]rime here [*in the ME*], is almost non-existent compared to SA.’ (Participant 07, male, private sector) ‘… [*A*] lot of us need to migrate and to provide a bit of safety and lifestyle for our families …’ (Participant 06, female, private sector) 3.2. Having enough time and money for interests other than work. ‘… I could go abroad and have time, particular time set aside to pursue personal growth in terms of postgraduate studies and while I am earning a reasonable salary …’ (Participant 01, male, public sector) 3.3. A more reasonable workload leading to a better work or life balance. ‘[*T*]he workload is a little bit less …’ (Participant 05, male, private sector) ‘You get to enjoy when you can, you can enjoy your work. And if you can bring your family, then it also brings a lot of relief in terms of stress …’ (Participant 07, male, private sector)
4. Poor management at the workplace	4.1. Poor organisational leadership and management skills. ‘They [*my managers*] are not innovative …’ (Participant 03, male, public sector) ‘There’s a bit of dictatorship …’ (Participant 03, male, public sector) 4.2. Lack of support from organisational management. ‘I was not getting the necessary support from management above me to enable me to operate …’ (Participant 01, male, public sector) ‘You don’t have equipment to do your job optimally. You address that with management, they simple doesn’t seem to be doing anything with regards to that.’ (Participant 04, male, public sector) 4.3. No organisational and career advancement opportunities. ‘[*P*]ositions are blocked by people who have little to no managerial skills …’ (Participant 08, male, public sector) ‘[*T*]he leadership has not changed in my time, it is still not changed. You know the CEOs are still the CEOs, the senior managers are still the same senior managers …’ (Participant 05, male, private sector) 4.4 Not being valued as a skilled clinician and professional. ‘[*W*]e have managers that are AEAs who have no clinical understanding or context or what your role in the organisation is, what you your purpose is or what you can do.’ (Participant 09, male, public sector) ‘[*T*]he understanding of the capabilities of an ECP when it comes to management. They downplay in terms of what equipment you should be getting.’ (Participant 04, male, public sector)	-
		-
		-
		-

*Source:* Mthombeni, S.I., 2024, ‘Factors influencing the migration of South African emergency care practitioners to the Middle East’, Masters thesis, University of Johannesburg, Johannesburg, viewed n.d., from https://hdl.handle.net/10210/511825

SA, South Africa; ME, Middle East; EMS, Emergency Medical Services; ECP, Emergency Care Practitioner.

## Discussion

### Demographics

The 55 responses analysed suggest that at minimum, 8% of the ECPs registered with the HPCSA were working outside of SA in the ME for at least one year or more at the time of the study. This proportion would have surely been higher if our study included all other ECPs that were working out of SA borders. The majority of our respondents were male and had migrated to the ME with their families in an attempt to provide comfort and safety. Govender et al. (2012) also noted similar findings in their study about the migration of paramedics from SA and this also adds to support the view that historically men with family are more likely to migrate in search of better environments for their children (Ajith 2021; Govender et al. 2012). Interestingly, there was a greater representation of blacks in our sample compared to other studies that have investigated the migration of South African HCWs (Bezuidenhout et al. 2009; Bidwell et al. 2014; Govender et al. 2012; Reardon & George 2014). Reasons for this are unclear; however, it is speculated that these results may suggest that racial inequality in the field of SA pre-hospital education and training that has historically been an issue is being positively addressed, and more black people are now afforded the same opportunities at obtaining advanced-level qualifications as their white counterparts. In all, 63% of our respondents were between the ages of 25 and 35 years and had worked an average of 3 years in SA as ECPs after qualifying. This shows that the majority of ECPs do not stay long in SA before they start to seek employment opportunities internationally.

### Push and pull factors

#### Financial

Finances featured as both the leading push and pull factor with all our participants citing the low wages that they were getting in SA as the most significant influencer of their decision to start looking for international employment. Many similar studies investigating the migration of HCWs from LMICs to high-income countries (HICs) also found that money is the biggest motivator for economic migration (Asadi et al. 2018; Govender et al. 2012; Reardon & George 2014; Toyin-Thomas et al. 2023). The effect of lower wages may be greater in the context of emerging professions that historically are not as well paid as more established professions. The South African EMS context is such that, being in the EMS is often viewed by the public as a ‘privilege’ and that it is one of the more rewarding and ‘better-paying’ jobs within the country. This may be premised on a view that having obtained a professional bachelor’s degree from a university which leads to work in a demanding and stressful clinical environment one’s take-home pay should be able to support a ‘comfortable’ middle-class existence in SA. However, in reality, this appears not to always be the case. Rather, it would seem that, while our participants were working in SA, they were experiencing significant financial hardship caused by what they deemed to be insufficient ‘take-home’ pay. Their financial hardship in SA was further complicated by a declining economy and an ever-increasing cost of day-to-day living.

It is a fact that economic conditions in SA have been on the decline for many years now, and the cost of living in SA is getting higher and higher (Arndt et al. 2020; Mostert 2023; Redl 2015). Our participants’ inability to make ends meet on their ECP take-home salaries in SA appears to have culminated in them reaching the point where they were financially ‘stressed’. This, together with high-income tax and other salary deductions played a significant role in them deciding to seek employment in the ME. Similar findings are reported by other studies that also found that many HCWs who have migrated from LMICs were simply tired of enduring economic instability and uncertainty in their home countries, and they felt that seeking economical refuge from HIC would provide them and their families with better future prospects (Hajian et al. 2020; Konlan, Lee & Damiran 2023).

The effect of taxation also came across as a factor with many of our participants sharing a view that, while local salaries may at face value appear market-related and adequate, this is before taxation, and their take-home pay after taxation was simply insufficient for them to sustain a suitable quality of life. Such findings are consistent with other studies that reported that many SA-qualified doctors left the country because they were frustrated with the unfair income tax system in SA (George et al. 2013; Mlambo & Adetiba 2017). Other studies have therefore suggested that better pay and financial incentives such as tax relief and allowances can become important motivating factors for the retention of skilled HCWs in LMICs such as SA (Akintola & Chikoko 2016; Li et al. 2019). Even though only a small portion of our respondents were found to have worked in the semi-rural and rural areas, Henderson and Tulloch (2008) do confirm that many LMICs have adopted similar financial incentives for such locations as a means of attracting and retaining HCWs specifically within the public sector. Participants in our study also reported that their SA employers were made aware that they were not able to make ends meet on their SA salaries, yet they reportedly failed to engage them on this aspect.

Similarly, negative comments were expressed about the failure of the national government to implement an appropriate Occupation Specific Dispensation (OSD) policy that speaks to the professional qualification structures in the EMS sector. Participants in our study viewed the current OSD scales that are used to compensate EMS providers as outdated, and it fails to adequately recognise the skill and complexity of the ECP qualification.

It is true that the salary scales that are currently offered to ECPs in SA are not comparable to other healthcare professions with similar undergraduate qualifications such as doctors and nurses, many of whom are paid over and above their basic salary based on a recognition of their scarce skills and areas of specialisation. Those participants that worked in the private sector prior to leaving SA also revealed that they felt they were left with little room for negotiating a better salary as their organisations had started benchmarking offers based on the current government OSD scales. While this has been noted by other studies, these tend to be discipline-specific and contextual. About a decade ago, Labonte et al. (2015) reported that the introduction of the OSD and other migration policies did in fact result in reducing the local and international migration of certain cadres of HCWs from SA; these, however, seem to be no longer effective particularly in the pre-hospital sector as international migration of all qualifications is forever on the rise.

#### The desire to gain international experience

Surprisingly, about two-thirds of our respondents indicated that the desire to gain international experience and live a better quality of life in the ME was another factor that played a huge role for them in choosing the ME as their employment destination. Literature suggests that as much as 86% of HCWs migrate to HICs because of a ‘need’ for exploration, international training and experience (Vujicic et al. 2004). In the context of our study, this need is easily fulfilled by the ‘abundance’ of international opportunities that are available for South African ECPs in the ME. This seemed to have been coupled to an idea that having international experience gives one a future advantage in their careers.

The prospect of living a balanced, safe and better quality of life in the ME while also being able to better provide for their families was also seen by our participants as a positive factor for their move. The presence of friends who were already living and providing positive report about the ME was also found to have had a significant influence on our participants’ decision. This certainly does not sound to be in favour of the SA EMS as it might have a snowball effect that might potentially see more ECPs migrating to the ME.

Even though only a quarter of our respondents indicated that they will continue to work for EMS when they eventually return to SA, the findings, however, suggested that a large proportion (63%) of these respondents had chosen the ME as their employment destination with the idea of being exposed to, and learning from other more developed EMS systems which would allow to better contribute to the development and progression of the local EMS system should they return to SA. Motlhatlhedi and Nkomazana (2018) support such views reporting that HCWs often return home with a strong work ethic and are able to transfer the clinical skills and knowledge acquired while working abroad. Taking this into account, we suggest that local EMS could consider travel incentives and staff exchange programmes as a mutually beneficial way to fulfil this need for international exposure and also bring new skills back to the service.

#### Working conditions and environment

High workloads and unsafe working conditions in SA EMS were other significant factors that influenced ECPs to start looking for employment overseas. Forty-six percent of our respondents indicated they were unhappy with the SA EMS working environment. This is consistent with other studies that have also noted that poor and unsafe working conditions are one of the strong push factors of HCWs migration from LMICs (Govender et al. 2012; Konlan et al. 2023; Saluja et al. 2020).

An apparently ‘unbearable workload’ while in SA and the desire for an environment with a better work or life balance also played a significant role in our participants’ decision to leave SA. Many participants indicated that they felt overworked, burnt out and that they had little time for their families while they were working operationally in SA. One of the reasons they advanced for this was a shortage of operational ECPs. In this regard, several studies have highlighted the importance of having sufficient human resource capacity with adequate skills and expertise for the success of an organisation (AlShammari, Jennings & Williams 2018; Joshi et al. 2023; World Health Organization 2016). Available literature on pre-hospital healthcare in SA also confirms that there remains a shortage of ALS providers and in particular ECPs to cater to the needs of the South African community (Govender, Grainger & Naidoo 2013; Hackland & Stein 2011; Iwu 2013). Some of the reasons that have been attributed to the shortage of ECPs include the low number of graduates from local universities (currently there are only four that offer the ECP qualification), a lack of funded posts to accommodate graduates coupled with the continuous poaching and recruitment of ECPs by EMS organisations in HICs.

The shortage of ECPs means that unlike more developed EMS systems, in SA, ECPs are commonly expected to work alone on an emergency vehicle. A shortage of staff and vehicles also means that ECPs in SA commonly have to care for critically ill and injured patients for lengthy periods of time on their own prior to the arrival of an ambulance. Aside from the obvious patient care and quality concerns, the practice of placing a single ECP onto a response vehicle needs to be critically evaluated as this also leads to a number of additional safety and security concerns.

The above-stated context is concerning, as is the shelf life of an operational ECP in SA which is often only between 3 to 5 years. Another work-related factor that appears to have played a significant role in driving our participants to migrate was the issue of workplace violence. Our participants revealed that they consistently feared for their lives while they were working in SA. This stress was also affecting their immediate families as they were constantly worried if their loved ones would come home from shift. Also shared were our participants’ experiences of being personally robbed and/or attacked while engaged in operational duties and that they did not feel that the community members, EMS managers and the police services in SA were doing enough to protect them.

Workplace violence against EMS providers is not just a South African phenomenon; rather, this is an issue that is increasing globally (Maguire 2018; Murray et al. 2020; Shabanikiya et al. 2021). A literature review by Murray et al. (2020) reported that as much as 93% of EMS providers are exposed to some form of workplace violence, whether it be verbal and/or physical abuse from colleagues, patients, patients’ families and members of the community. Studies from SA confirm that, in the past couple of years, the SA pre-hospital sector has indeed experienced a steep increase in the number of attacks on emergency care providers (Khoza, Sibiya & Mshunqane 2022; Vincent-Lambert & Westwood 2019). This has reached a point where certain areas have been marked as hotspots or ‘red’ zones where ambulances cannot enter without police escorts. Vincent-Lambert and Westwood (2019) identified a need for a hostile environment awareness training for undergraduate ECP curriculum to sensitise, prepare and better equip them for the operational environment in SA. Other research has since highlighted that improving working conditions and safety at the workplace while also investing in career and organisational development are important aspects in retaining skilled workers within an organisation (Mlambo & Adetiba 2017; Mumbauer et al. 2021; Tomblin Murphy et al. 2016).

Taking the preceding into account and the focus of our study, while we acknowledge that no country is perfect, the ME too has safety and security concerns. However, the countries that the majority of our respondents have migrated to in the ME appear to have far lower levels of contact and violent crime than those in SA. This may be why our participants reported that they were content with their decision to migrate to the ME as they no longer had fears of being robbed or losing their lives while on duty.

#### Poor management

Effective professional managers play a significant role in keeping employees happy and retaining them within an organisation (Conroy et al. 2023; De Vries et al. 2023). A number of our participants shared experiences of what they saw as poor leadership and ‘bad’ management that drove them to leave SA. Participants apparently became frustrated by not being heard, undermined and undervalued by their managers, many of whom they saw as ‘old’, set in their ways and yet less qualified than them. Literature also supports the view that many of the local EMS managers held an intermediate clinical qualification without formal leadership and/or management qualifications and are resistant to change and adopting new ideas (Naidoo, Lowies & Pillay 2014).

In keeping with the foregoing, the need for better leadership and strategic management skills in EMS environments is not isolated to SA but appears to be a global phenomenon. A study on multi-national EMS providers conducted in the KSA found that leadership and management skills are not routinely included in EMS training programmes but learned informally from other agencies or mentors (Leggio 2014). Low levels of emotional intelligence and under-developed people management skills have also been identified as one of the barriers that is preventing the progression and expansion of EMS in LMICs (Nielsen et al. 2012). Similarly, a number of our participants also shared a view that many of their managers in SA possessed poor communication skills and adopted an authoritarian managerial leadership style that created frustration, fear and unhappiness among the operational staff which ultimately led to job dissatisfaction, particularly amongst ECPs.

A study by Hackland and Stein (2011) found similar experiences, one of their findings being that one of the main reasons for ALS providers to leave the operational environment in SA was poor management and leadership styles within their organisations. This suggests that, if we are to prevent more ECPs from leaving the country, the current EMS management structures have to change, and people who are better qualified and/or formally trained in leadership and management should be appointed as operational managers rather than those who have simply been in the service for a long period of time.

### Limitations

The low number of the responses was because this research only included SA-qualified ECPs that were located in the region of the ME. Therefore, these results cannot be generalised to the entire SA EMS population that have migrated from SA to other regions. Furthermore, even though the respondents had an option to add other factors that may have influenced them to migrate, we acknowledge that the pre-determined of the push and pull factors in our questionnaire may have posed some limitations in determining other factors such as the lack of affirmative action from the organisation and having opportunity to travel and explore the world (both identified and added by our respondents). We also concede that this study was limited to describing what we found as opposed to drawing correlations between our sample and the national demographics in SA. Further studies are needed from other regions.

## Conclusion

The SA EMS is experiencing a huge shortage of highly skilled pre-hospital providers which is exacerbated by aggressive recruitment from international EMS organisations and the continuous migration of ECPs. Higher wages, a safer and generally better working and living environment renders the ME a sought-after destination for SA-qualified ECPs. Significant work has to be done by both the public and private sectors to address the OSD, improve salaries, working conditions and overall job satisfaction experienced by ECPs if we are to stem the ongoing exodus of this cadre of healthcare professional.
